# Antihypertensive Treatment in Kidney Transplant Recipients—A Current Single Center Experience

**DOI:** 10.3390/jcm9123969

**Published:** 2020-12-07

**Authors:** Ulrich Jehn, Katharina Schütte-Nütgen, Markus Strauss, Jan Kunert, Hermann Pavenstädt, Gerold Thölking, Barbara Suwelack, Stefan Reuter

**Affiliations:** 1 Department of Medicine D, Division of General Internal Medicine, Nephrology and Rheumatology, University Hospital of Münster, 48149 Münster, Germany; Ulrich.jehn@ukmuenster.de (U.J.); katharina.schuette-nuetgen@ukmuenster.de (K.S.-N.); jbkunert@t-online.de (J.K.); hermann.pavenstaedt@ukmuenster.de (H.P.); gerold.thoelking@ukmuenster.de (G.T.); Barbara.Suwelack@ukmuenster.de (B.S.); 2 Department of Medicine C, Division of Cardiology and Angiology, University Hospital of Münster, 48149 Münster, Germany; markus.strauss@ukmuenster.de

**Keywords:** kidney transplantation, post-transplant care, arterial hypertension, antihypertensives, allograft failure

## Abstract

Arterial hypertension affects the survival of the kidney graft and the cardiovascular morbidity and mortality of the recipient after kidney transplantation (KTx). Thus, antihypertensive treatment is necessary for a vast majority of these patients. Long-term data on antihypertensive drugs and their effects on allograft function after KTx is still limited, and further investigation is required. We retrospectively analyzed a cohort of 854 recipients who received a kidney transplant at our transplant center between 2007 and 2015 with regard to antihypertensive treatment and its influence on graft function and survival. 1-y after KTx, 95.3% patients were treated with antihypertensive therapy. Of these, 38.6% received mono- or dual-drug therapy, 38.0% received three to four drugs and 8.1% were on a regimen of ≥5 drugs. Beta-blockers were the most frequently used antihypertensive agents (68.1%). Neither the use of angiotensin-converting enzyme inhibitor/angiotensin receptor blockers (51.9%) and calcium channel blockers (51.5%), nor the use the use of loop diuretics (38.7%) affected allograft survival. Arterial hypertension and the number of antihypertensive agents were associated with unfavorable allograft outcomes (each *p* < 0.001). In addition to the well-known risk factors of cold ischemic time and acute rejection episodes, the number of antihypertensive drugs after one year, which reflects the severity of hypertension, is a strong predictor of unfavorable allograft survival.

## 1. Introduction

Cardiovascular disease (CVD) is the leading cause of mortality and morbidity in kidney transplant (KTx) recipients [1]. Thus, arterial hypertension (AHT) is a significant risk factor that affects a large proportion of this patient population (50–80% of adult recipients) [2]. Increased systolic and diastolic blood pressure are associated with allograft failure [3]. However, it has been shown that target systolic blood pressures below 120 mmHg in KTx recipients, as recommended for patients at a high risk for cardiovascular events [4], does not negatively affect allograft function [5], favoring the need for strict blood pressure control in KTx recipients.

Most kidney transplant recipients have a long-term history of chronic kidney disease (CKD) associated with vascular pathologies leading to elevated systolic blood pressure (SBP) and increased pule pressure [6]. After KTx, additional factors occur that are capable of inducing AHT. First, the unavoidable use of immunosuppressants, especially calcineurin inhibitors (CNIs), and steroids is implied to induce or promote AHT [7]. Second, volume overload is a common issue in patients with renal (allograft) insufficiency, and induces elevated blood pressure [8]. Third, renal insufficiency following KTx can also cause AHT, since the allograft and also the diseased autologous kidneys can trigger AHT, mainly via altered sodium regulation and the activation of the renin–angiotensin–aldosterone system (RAAS) [9]. Fourth, transplant renal artery stenosis accounts for 1–5% of cases of post-transplant AHT [10]. Fifth, post-transplant weight gain and obesity are common and lead to a significantly increased prevalence of the metabolic syndrome, both of which favor AHT [11].

In KTx recipients, donor factors such as the donor’s pre-existing AHT, higher donor age, poor allograft quality and genetic donor factors can also influence the recipient’s blood pressures [2].

To date, there have not been randomized controlled trials on optimal blood pressure limits or favorable treatment strategies in patients after KTx [2]. Therefore, the Kidney Disease Improving Global Outcomes (KDIGO) and National Kidney Foundation guidelines for the management of AHT in patients with CKD are widely followed when AHT is treated in KTx recipients [12], targeting a blood pressure <130/80 mmHg.

Nevertheless, there are substantive differences in the treatment of non-transplant patients, as, for example, some of the factors mentioned above that contribute to AHT in KTx recipients are transplant-specific.

A recent cross-sectional study from Poland showed that beta-blockers were the most commonly used antihypertensive agents, followed by Calcium channel blockers (CCB), angiotensin-converting enzyme inhibitors (ACE-I)/Angiotensin-II-receptor-blockers (ARB), and diuretics [13]. The remaining drug classes, such as alpha1-receptor-antagonists, anti-sympathotonics and vasodilators are usually prescribed as reserve antihypertensives.

In CKD patients, the blockage of RAAS has been proved to slow down the progression of diabetic and non-diabetic (protein-rich) kidney disease [14,15]. Several years ago, we observed in our KTx cohort that the use of ACE-I/ARB and CCB promoted long-term graft survival in KTx recipients [16]. A recent meta-analysis of 71 randomized controlled trials by Pisano et al. also supports the preferred use of CCB, as they were found to improve graft function as well as graft survival [17]. In this meta-analysis, ACE-I and ARB treatment was associated with worsening effects on allograft function and a 3-fold higher risk for hyperkalaemia—nevertheless there is some evidence for improvement of graft survival regarding RAAS inhibitors [17].

To date, however, there is no data demonstrating an association between the use of beta-blockers or diuretics and the long-term outcome of kidney allografts and KTx recipients although both drug classes are widely used. The frequent co-incidence of different therapeutic indications especially for beta-blockers and/or diuretics in addition to the treatment of AHT in the KTx setting, e.g., treatment of congestive heart failure or atrial fibrillation, is a problematic pitfall in this matter [18,19]. Especially for loop diuretics, the evidence for their perioperative use in KTx is still controversial [20].

The present study was conducted in order to shed some light on the relationship between antihypertensive drugs and long-term outcomes for KTx recipients in the present era, taking into account that former studies are predominantly ten to twenty years old and KTx recipient outcome has changed rapidly within the last years [21]. Our analyses focused on how AHT treatment in the first year predicted the subsequent allograft survival.

## 2. Materials and Methods

### 2.1. Study Design and Population

We analyzed the data of 850 adult patients (follow-up of 4109.5 patient years, median follow-up 4.2 years) who were transplanted at our center between 1 January 2007 and 31 December 2015. The demographics and clinical characteristics of the patients were recorded at the time of KTx, and written informed consent was obtained from all patients to record their data. The data was taken from the patients’ files, and the personal information was made anonymous prior to the analysis. This study was performed in accordance with the Declaration of Helsinki and the International Conference on Harmonization of Good Clinical Practice guidelines and approved by the local ethics committee (Ethik Kommission der Ärztekammer Westfalen-Lippe und der Medizinischen Fakultät der Westfälischen Wilhelms-Universität, 2014-381-f-N). Induction therapy was chosen based on the immunological risks of the patients. The triple immunosuppressive standard regimen consisted of tacrolimus (6–10 ng/mL), mycophenolate mofetil and steroids (Table 1). Estimated glomerular filtration rate (eGFR) was calculated using the CKD–EPI formula [22].

### 2.2. Blood Pressure Monitoring and Antihypertensive Medication

Blood pressure was measured manually during the outpatient one-year routine follow-up according to the 2013 European Society of Hypertension (ESH)/European Society of Cardiology (ESC) Guidelines for the management of AHT [23] and the antihypertensive medication that each patient was taking at the time of the one-year follow-up was simultaneously recorded.

We categorized antihypertensive medication into ACE-I/ARB, CCB and diuretics, subclassified into loop diuretics, thiazids and aldosterone-antagonists, beta-receptor-blockers, alpha1-receptor-antagonists, anti-sympathotonics and vasodilators.

### 2.3. Statistical Analysis

The data was analyzed with IBM SPSS Statistics 26 (IBM Corp., Armonk, New York, USA). The results are expressed as a mean with standard deviation, median with interquartile range (IQR) or number/percent. The categorical parameters were analyzed by Fisher’s exact test and the chi-square test and continuous parameters were analyzed by Mann–Whitney U-test and Kruskal–Wallis test, respectively. A *p*-value below 0.05 was considered statistically noticeable.

Linear regression analysis was performed to test association between two continuous variables. The cumulative probability of developing allograft failure in the kidney transplant cohort was calculated by Kaplan–Meier analysis [24], and the curves were compared using the log-rank test. To evaluate the risk factors for the onset of allograft failure, we performed multivariable Cox regression analyses [25] to clarify whether beta-blockers or steroids are independent risk factors for reduced kidney allograft survival.

## 3. Results

### 3.1. Sample Characteristics

The baseline characteristics of the study population are presented in Table 1. Mean age at transplantation was 52.9 ± 14.2 years, 61.0% of the recipients were male and 26.9% received a living donor transplantation. A tacrolimus-based immunosuppressive triple regimen was used for 94.1% of patients, while 4.9% received cyclosporine (Table 1).

Of the initial 850 patients, 765 patients could be evaluated for antihypertensive medication after 1-y; 21 patients died with functioning allograft (2.5%), 37 patients (4.3%) developed terminal allograft failure and 27 patients (3.2%) were lost to follow-up during the first year.

In our patient cohort, beta-blockers were the most frequently used antihypertensive drug group one year after KTx, prescribed to 582 patients (68.1%). ACE-Is/ARBs were prescribed to 443 (51.9%), while 440 patients (51.5%) were treated with CCRBs and 375 (43.9%) with diuretics. Among them, loop diuretics were by far the most frequently prescribed (331, 38.8%), while 29 patients (3.4%) received a thiazide diuretic and 23 (2.7%) received an aldosterone antagonist. In 94 (11%) patients, anti-sympathotonic drugs were administered, together with at least three other antihypertensive drugs. The same applies to alpha1-receptor blockers and vasodilators, which were prescribed to 30 (3.5%) and 20 (2.3%) patients, respectively.

### 3.2. Clinical Characteristics of Patients and the Use of Antihypertensive Agents

Clinical outcome parameters are presented in Table 2. Patients who received a living donor KTx were treated with less antihypertensive agents (mean 2.1 ± 1.3) compared to recipients of post-mortal donations (mean 2.9 ± 1.4), (*p* < 0.001). Moreover, patients with a maximum of two antihypertensives were significantly younger (*p* < 0.001). Due to the higher percentage of living donations in younger patients, the cold ischemia time was significantly shorter (*p* < 0.001) in this group than in the group of older patients receiving three or more antihypertensives after one year.

### 3.3. Blood Pressure and Renal Allograft Function

Despite intensified antihypertensive medication, the systolic blood pressure was higher one year after KTx in patients with a higher number of prescribed antihypertensive agents (*p* < 0.001). In contrast, the mean diastolic blood pressure did not differ between the groups (*p* = 0.374). Clearly, the mean systolic blood pressure after one year was associated with impaired allograft function in terms of lower eGFR one (*p* < 0.001, r = −0.115), two (*p* < 0.001, r = −0.160) and five years after KTx (*p* < 0.001, r = −0.204). There was no association of diastolic blood pressure with eGFR after one (*p* = 0.085, r = 0.036), two (*p* = 0.970, r = −0.001) and five years (*p* = 0.923, r = 0.003).

### 3.4. Renal Allograft Function and Number of Antihypertensive Agents

**Figure 1:**
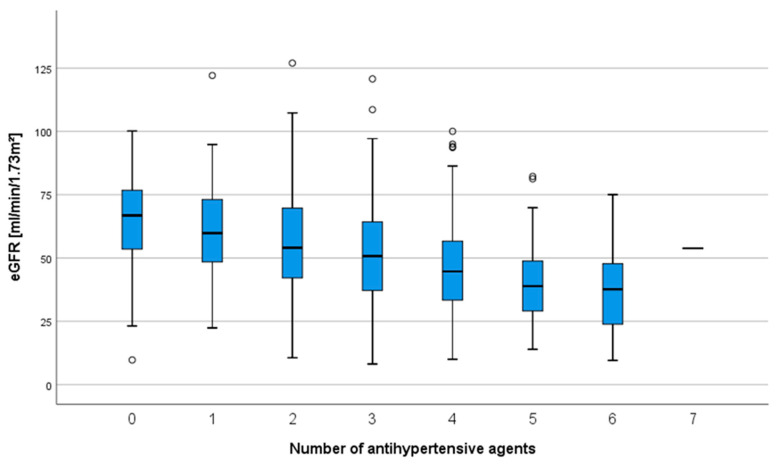
Estimated glomerular filtration rate eGFR after one year is strongly associated with the number of antihypertensive agents, Kruskal–Wallis test, *p* < 0.001.

Renal allograft function was strongly correlated with the number of antihypertensives in the study. After one year, patients without AHT treatment had a mean eGFR of 63.9 ± 19.4 mL/min/1.73 m2 while patients with six antihypertensives had a mean eGFR of 39.0 ± 17.2 mL/min/1.73 m2 (*p* < 0.001, Figure 1).

**Figure 2:**
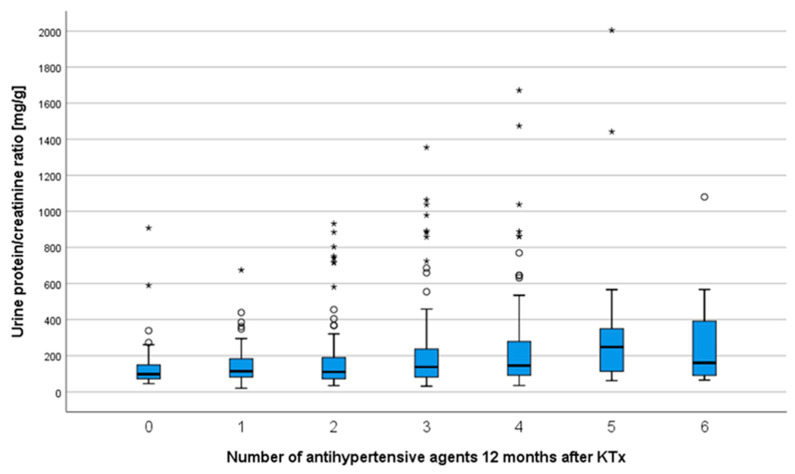
Proteinuria after one year increased noticeably as the number of antihypertensive agents increased; Kruskal–Wallis test, *p* < 0.001. * extreme outlier.

Similar results were found with regard to the number of antihypertensives and the urine protein/creatinine ratio (UPCR). After one year, patients without antihypertensives had a mean UPCR of 160 ± 181 mg/g creatinine, while those with six antihypertensives had a noticeably higher mean UPCR of 443 ± 696 mg/g creatinine (*p* < 0.001, Figure 2).

### 3.5. Antihypertensive Agents and Renal Allograft Function

In patients medicated with ACE-I/ARB, beta-blockers or CCB, eGFR and UPCR are comparable. In contrast, patients treated with a loop diuretic show reduced eGFR and elevated UPCR (Table 3).

### 3.6. Antihypertensive Agents and Allograft Survival

**Figure 3:**
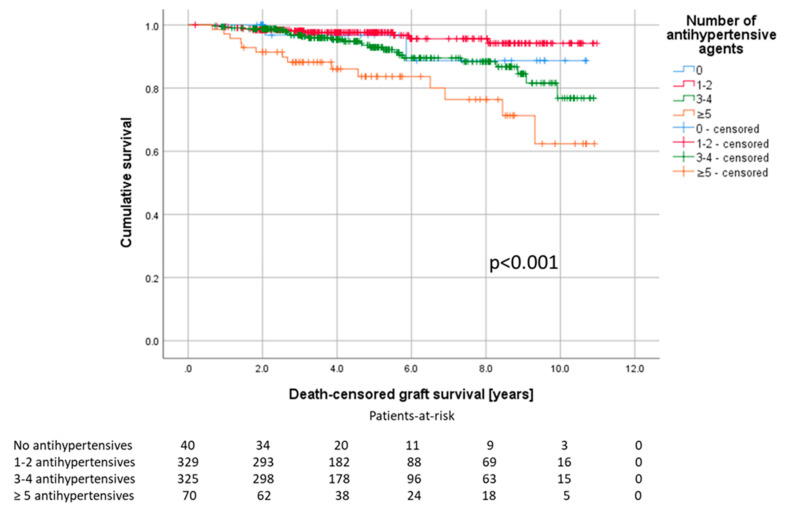
Patients on five or more antihypertensive agents show the shortest graft survival, Log-rank test, *p* < 0.001.

One year after KTx, the number of antihypertensive agents was associated with lower eGFR and higher proteinuria. The Kaplan–Meier analysis and log-rank test showed a shortened death-censored graft survival for patients on five or more different antihypertensive drugs (8.9 years, CI 8.0–9.8). Mean allograft survival for patients without antihypertensives was 10.0 years (CI 9.1–10.9), for patients with one or two antihypertensives 10.6 years (CI 10.4–10.8) and for patients with three or four antihypertensives 9.9 years (CI 9.6–10.3), (*p* < 0.001, Figure 3).

Recipients treated with beta-blockers had an inferior allograft survival compared to those who were not (10.0 years, CI 9.8–10.3 vs. 10.4 years, CI 10.1–10.7), (Log-rank, *p* = 0.022, Supplementary Figure S4A). Similarly, medication with loop diuretics was associated with graft survival shorter by 0.5 year (10.3 years, CI 10.1–10.6 vs. 9.8 years, CI 9.4–102), (*p* = 0.001, Supplementary Figure S4B). In contrast, the Kaplan–Meier analysis showed no differences in the kidney allograft survival for the use of ACE-I/ARB (*p* = 0.398, Supplementary Figure S4C) or CCB (*p* = 0.102, Supplementary Figure S4D). The reserve antihypertensive drug groups of vasodilators, anti-sympathotonics and α-receptor blockers are all associated with inferior allograft outcome (all *p* < 0.001).

### 3.7. The Number of Antihypertensive Drugs as Indicator for Death-Censored Kidney Allograft Failure

A multivariable Cox regression analysis was performed to assess whether the number of antihypertensive drugs or medication with loop diuretics or beta-blockers was associated with inferior allograft survival independent from other known risk factors. The analysis included several known risk factors for reduced allograft survival and the number of antihypertensives at 1-y after KTx, as loop diuretics were usually used as 3rd or 4th line therapy (Table 4). In addition to the cold ischemia time (*p* = 0.014, HR 1.104) and the occurrence of at least one acute rejection episode (any type of rejection) (*p* < 0.001, HR 4.428), Cox regression analysis confirmed the number of antihypertensive agents one year after transplantation as independently associated with death-censored allograft failure (*p* = 0.025, HR 1.525). Neither the use of beta-blockers (*p* = 0.820, HR 0.884) nor the use of loop diuretics (*p* = 0.404, HR 1.388) was independently associated with allograft failure.

### 3.8. The Development of Kidney Function as a Function of the Number of Antihypertensive Drugs

To further clarify whether antihypertensive drugs themselves may be harmful for allograft function, we studied the relation between the number of antihypertensive drugs after one year and the eGFR-change from year one to two and from year one to five. Overall, eGFR improved slightly during both periods. From year one to two, there were no significant differences between the groups (Table 5). From year one to five, only the differences between patients without antihypertensives and those with 1–2 (*p* = 0.023) and 3–4 (*p* = 0.015) were significant.

## 4. Discussion

AHT is one of the main risk factors for cardiovascular events after KTx [2]. Since prospective studies on the impact of different antihypertensives after KTx are scarce, further data on this important topic is needed.

In the present study, we analyzed data from our transplant center and divided patients into four groups based on the number of different antihypertensive drugs. The distribution of main antihypertensives used in our cohort was comparable to that of other transplant studies [13]. Our data showed that the number of different antihypertensives of KTx recipients is a strong predictor of poor allograft function and survival. Nevertheless, the patients taking an extended number of antihypertensive substance classes showed poorly controlled AHT. This subgroup also showed increased mean systolic blood pressure of 142 ± 16 mmHg, according to the target blood pressure recommended by KDIGO for CKD patients: <130 mmHg [26]. As KTx recipients frequently experience adverse effects by polypharmacy [27] and several drug interactions [28], non-adherence might contribute to these findings [29].

As expected, KTx recipients with severe hypertension were older, received less frequently living donations, and had a longer cold ischemic time associated with a higher rate of delayed graft function (DGF). In accordance with the literature, these factors can be regarded as confounders for inferior allograft outcome and have an impact on the development of AHT [2,6].

Cox regression analysis confirmed that the number of antihypertensive agents prescribed to a patient after one year is independently related to death-censored allograft failure (*p* = 0.025), whereas the number of antihypertensives usually reflects the severity of AHT. Interestingly, our data show that a higher number of antihypertensive drugs administered to patients (three or more) compared to patients treated with two or less antihypertensives is not associated with a higher eGFR-loss over time (Table 5). Although, this analysis does not consider patients with allograft loss, it suggests that worse allograft or recipient characteristics are more likely to determine arterial hypertension than antihypertensive drugs themselves affect allograft function.

In terms of renal function, KTx recipients who received loop diuretics, usually administered as third- or fourth line medication (Table 2), showed reduced eGFR values and higher levels of UPCR (Table 3). This also indicates that impaired allograft function promotes hypertension. Since almost 40% of the patients in our cohort took loop diuretics one year after KTx, these patients represent a significant high-risk group for allograft loss, which requires increased vigilance by the attending physicians with this patient group. Unfortunately, there is at present no available study data on the role of (loop) diuretic application after KTx. In the perioperative period of KTx, the use of loop diuretics to increase diuresis and prevent DGF or dialysis has been evaluated in different studies, but there is no conclusive evidence to support its use in this context [20].

In CKD patients, Ishikawa et al. observed that the intake of loop diuretics was associated with sarcopenia [30]. Nevertheless, a literature review revealed cardioprotective effects for low-dose loop diuretics to some extent, with a reduction in hospitalization rate during the pre-dialysis and hemodialysis stages [31,32]. In the treatment of acute kidney injury (AKI), loop diuretics have no beneficial effect on the renal prognosis [33]. Since thiazids and aldosterone antagonists were only prescribed in a few patients (3.4% and 2.7%), as they are contraindicated or ineffective in patients with CKD stages 4–5, we did not further analyze these agents.

The use of beta-blockers was also associated with inferior allograft survival in the Kaplan–Meier analysis. However, multivariable Cox regression could not confirm beta-blockers as a risk factor for reduced death-censored allograft survival. Although they are the most frequently used antihypertensive agents after KTx in our cohort as well as in others [13,33], data on their influence on the outcomes of renal allograft outcome is scarce. Their frequent usage might be related to a high percentage of patients with chronic heart failure and/or arrhythmias as atrial fibrillation in the KTx patient collective [18,19].

With regard to the results of a former study from our transplant center [16], we could not observe a beneficial effect of ACEI/ARB or CCB on allograft survival. Neither of the drug classes were associated with increased allograft survival in our cohort (Figure S4C,D). These observations require further elucidation because the reno-protective effects of both drug classes are known. Cross et al. reported that CCB use is associated with higher GFR values as it mitigates vasoconstrictive CNI effects in KTx recipients [34]. One benefit of RAAS blockade in non-transplant CKD patients could be clearly proved [14,35]. In KTx recipients, RAAS blockers seem as effective as other agents in lowering BP [36]. Regarding allograft and patient survival, most studies were unable to show a beneficial effect of ACEI/ARB [37].

One explanation for these results could be that patients treated with ACEI/ARB and/or CCB for AHT—a proven risk factor for poor allograft survival—are compared to patients without hypertension. Therefore, comparable allograft survival in these AHT patients may potentially be interpreted as a beneficial effect of these drugs.

Our study has several limitations. Since it is a retrospective analysis, it possesses only a hypothesis-generating character. In addition, the study design does not allow the distinguishing of direct effects of antihypertensive drugs on allograft function from specific factors that may be indicative, such as fluid overload due to reduced diuresis or congestive heart failure, which in turn is associated with poor graft function [38].

In summary, this study provides a current overview of the association between antihypertensive therapy and allograft outcome after KTx. Arterial hypertension, the severity of which reflects the number of antihypertensive drugs prescribed to a patient, remains one of the major factors in allograft failure. As our analyses suggest, allograft and/or recipient specific factors that cause AHT, rather than antihypertensive treatment itself, determine the increased risk for allograft failure in patients with advanced antihypertensive medication. Since AHT manifests in a vast majority of KTx patients prospective controlled trials are necessary the further investigate these findings. We must raise the awareness of transplant physicians with regard to monitoring and pharmacological treatment of AHT as it is an important risk factor for chronic dysfunction and failure of allografts, which can be—at least partially—influenced. One should keep in mind that KTx recipients receiving ≥3 different antihypertensive drugs are patients at risk who deserve special attention.

## Figures and Tables

**Table 1:** Patients’ demographics and clinical characteristics at transplantation.

Variable	All	No Antihypertensive Medication *	1–2 Antihypertensive Drugs *	3–4 Antihypertensive Drugs *	5+ Antihypertensive Drugs *	*p* Value
**Patients (n)**	850	40 (4.7%)	330 (38.6%)	325 (38.0%)	70 (8.1%)	
Age at KTx * (years)	52.9 ± 14.2	41.7 ± 13.2	49.8 ± 13.9	55.2 ± 13.3	59.3 ± 11.6	**<0.001 ^a^**
**Sex, male (n)**	521 (61.0%)	14 (35.0%)	191 (58.1%)	203 (62.3%)	53 (75.7%)	
**Mismatch-HLA-A**						**0.582 ^b^**
**none**	297 (34.8%)	12 (30.0%)	122 (37.1%)\	112 (34.4%)	21 (30.0%)	
**1**	402 (47.1%)	23 (57.5%)	147 (44.7%)	158 (48.5%)	32 (45.7%)	
**2**	148 (17.3%)	5 (12.5%)	59 (17.9%)	52 (16.0%)	16 (22.9%)	
**Mismatch-HLA-B**						**0.852 ^b^**
**none**	189 (22.1%)	7 (17.5%)	70 (21.3%)	46 (23.3%)	16 (22.9%)	
**1**	404 (47.7%)	19 (47.5%)	168 (51.1%)	149 (45.7%)	32 (45.7%)	
**2**	251 (29.4%)	14 (35.0%)	90 (27.4%)	97 (29.8%)	21 (30.0%)	
**mismatch-HLA-DR**						**0.012 ^b^**
**none**	221 (25.9%)	8 (20.0%)	75 (22.8%)	94 (28.8%)	23 (32.9%)	
**1**	397 (46.5%)	22 (55.0%)	173 (52.6%)	150 (46.0%)	20 (28.6%)	
**2**	229 (26.8%)	10 (25.0%)	80 (24.3%)	78 (23.9%)	26 (37.1%)	
**PRA%**	5.4 ± 17.7	5.7 ± 16.8	6.2 ± 19.5	5.2 ± 17.0	1.5 ± 8.9	**0.165 ^a^**
Living donor Tx * (n)	230 (26.9%)	22 (55.0%)	125 (38.0%)	66 (20.2%)	9 (12.9%)	**<0.001 ^b^**
**Cold ischemia time (hours)**	8.7 ± 5.2	5.8 ± 4.5	8.0 ± 5.3	9.2 ± 5.0	9.5 ± 5.0	**<0.001 ^a^**
Dialysis prior to Tx * (n)	795 (93.1%)	34 (85.0%)	296 (90.0%)	314 (96.3%)	67 (95.7%)	**0.001 ^b^**
Previous Tx* (n)	113 (13.2%)	6 (15.0%)	46 (14.0%)	43 (13.2%)	4 (5.7%)	**0.346 ^b^**
**CMV mismatch D/R**						**0.130 ^c^**
D^−^/R^-^	142 (16.6%)	9 (22.5%)	68 (20.7%)	39 (12.0%)	11 (15.7%)	
D^−^/R^+^	147 (17.2%)	6 (15.0%)	54 (16.4%)	62 (19.0%)	9 (12.9%)	
D^+^/R^−^	203 (23.8%)	9 (22.5%)	81 (24.6%)	75 (23.0%)	21 (30.0%)	
D^+^/R^+^	354 (41.5%)	16 (40.0%)	123 (37.4%)	148 (45.3%)	29 (41.4%)	
**Warm ischemia period (minutes)**	33 ± 8	30 ± 8	33 ± 9	34 ± 8	34 ± 8	**0.127 ^a^**
**Time on dialysis (months)**	57.7 ± 40.8	52.1 ± 45.1	55.6 ± 45.3	58.4 ± 37.0	61.7 ± 35.3	**0.164 ^a^**
**Initial steroid use**	844 (98.8%)	38 (%)	315 (95.5%)	323 (99.4%)	(%)	**0.107 ^b^**
Initial MMF* use	824 (96.5%)	40 (100%)	315 (95.5%)	315 (97.2%)	69 (98.6%)	**0.425 ^b^**
Initial CyA* use	42 (4.9%)	0 (0%)	15 (4.5%)	19 (5.8%)	4 (5.7%)	**0.325 ^b^**
**Initial tacrolimus use (n)**	804 (94.1%)	40 (100%)	310 (93.9%)	305 (93.8%)	66 (94.3%)	**0.397 ^b^**
Initial mTOR * inhibitor use(n)	26 (3.0%)	0 (0%)	11 (3.3%)	11 (3.4%)	1 (1.4%)	**0.568 ^b^**
**Diagnosis of ESRD, (n)**						**0.001 ^c^**
**Hypertension**	74 (8.7%)	1 (2.5%)	24 (7.3%)	36 (11.0%)	6 (8.6%)	
**Diabetes mellitus**	54 (6.3%)	3 (7.5%)	14 (4.3%)	22 (6.7%)	5 (7.1%)	
**Polycystic kidney disease**	127 (14.9%)	3 (7.5%)	57 (17.3%)	43 (13.2%)	10 (14.3%)	
**Obstructive nephropathy**	39 (4.6%)	3 (7.5%)	20 (6.1%)	14 (4.3%)	1 (1.4%)	
**Glomerulonephritis**	271 (31.7%)	7 (17.5%)	113 (34.3%)	106 (32.5%)	26 (37.1%)	
**FSGS**	36 (4.2%)	0 (0%)	9 (2.7%)	16 (4.9%)	5 (7.1%)	
**Interstitial nephritis**	45 (5.3%)	7 (17.5%)	14 (4.3%)	14 (4.3%)	4 (5.7%)	
**Vasculitis**	21 (2.5%)	0 (0%)	4 (1.2%)	11 (3.4%)	4 (5.7%)	
**Other**	110 (12.9%)	12 (30.0%)	43 (13.1%)	37 (11.3%)	3 (4.3%)	
**Unknown**	73 (8.5%)	4 (10.0%)	29 (8.8%)	26 (8.0%)	6 (8.6%)	

^a^ Kruskal–Wallis test, ^b^ Fisher’s exact test, ^c^ Chi-square test. * one year after transplantation. Abbreviations: CMV: cytomegalovirus; Tx: transplantation; HLA: human leukocyte antigen; PRA: panel reactive antibodies; MMF: mycophenolate mofetil; CyA: cyclosporine A; mTOR: mechanistic target of rapamycin; ESRD: end-stage renal disease; FSGS: focal segmental glomerulosclerosis. Bold: main variables and *p*-values.

**Table 2:** Patients’ clinical outcome parameters.

Variable	All	No Antihypertensive Drugs	1–2 Antihypertensive Drugs	3–4 Antihypertensive Drugs	≥5 Antihypertensive Drugs	*p* Value
**Mean syst. BP after one year (mmHg)**	135 ± 17	125 ± 24	133 ± 15	136 ± 17	142 ± 16	**<0.001 ^a^**
**Mean diast. BP after one year (mmHg)**	82 ± 11	81 ± 13	82± 11	81 ± 12	80 ± 12	**0.374 ^a^**
**DGF (*n*)**	208 (24.4%)	4 (10%)	52 (15.8%)	84 (25.8%)	22 (31.4%)	**0.001 ^b^**
**CMV DNAemia**	265 (31.0%)	12 (30.0%)	94 (28.6%)	123 (37.3%)	25 (35.7%)	**0.101 ^b^**
**BKV DNAemia**	182 (21.3%)	8 (20.0%)	75 (22.8%)	72 (22.1%)	18 (25.7%)	**0.736 ^b^**
**eGFR at year 1 (mL/min/1.73 m^2^)**	52.6 ± 20.2	63.9 ± 19.4	57.4 ± 19.7	49.2 ± 19.5	40.5 ± 16.2	**<0.001 ^a^**
**eGFR at year 2 (mL/min/1.73 m^2^)**	53.1 ± 20.9	65.9 ± 23.1	57.4 ± 20.1	49.6 ± 20.5	43.0 ± 17.6	**<0.001 ^a^**
**eGFR at year 5 (mL/min/1.73 m^2^)**	52.5 ± 21.8	56.0 ± 27.2	57.9 ± 22.4	49.9 ± 20.6	37.5 ± 11.2	**<0.001 ^a^**
**UPCR at year 1 mg/g crea**	283 ± 732	160 ± 181	183 ± 306	352 ± 874	549 ± 1410	**<0.001 ^a^**
**UPCR at year 2 mg/g crea**	260 ± 517	217 ± 271	196 ± 489	286 ± 465	481 ± 857	**0.008 ^a^**
**UPCR at year 5 (mg/g crea)**	323 ± 672	272 ± 272	150 ± 96	553 ± 1088	489 ± 487	**0.102 ^a^**
**Rejection yes (*n*)**	257 (30.1%)	10 (25%)	97 (29.5%)	101 (31.0%)	26 (37.1%)	**0.607 ^b^**
**ACE-I/ARB use**	443 (51.9%)	-	117 (35.6%)	261 (80.0%)	65 (92.8%)	**<0.001** **^b^**
**Calcium-channel blocker use**	440 (51.5%)	-	108 (32.8%)	264 (81.0%)	68 (97.1%)	**<0.001** **^b^**
**Beta-blocker use**	582 (68.1%)	-	217 (66.0%)	296 (90.8%)	69 (98.6%)	**<0.001** **^b^**
**Diuretic use**	375 (43.9%)	-	89 (27.0%)	219 (67.2%)	67 (95.7%)	**<0.001** **^b^**
**Antisympathotonic use**	94 (11.0%)	-	0 (0%)	42 (12.9%)	52 (74.3%)	**<0.001** **^b^**
**Vasodilator use**	20 (2.3%)	-	0 (0%)	8 (2.5%)	12 (17.1%)	**<0.001** **^b^**
**Alpha1-receptor blocker use**	30 (3.5%)	-	0 (0%)	9 (2.8%)	21 (30.0%)	**<0.001** **^b^**

^a^ Kruskal–Wallis test, ^b^ Fishers exact test. Abbreviations: DGF: delayed graft function; eGFR: estimated glomerular filtration rate; UPCR: urine protein/creatinine ratio; NODAT: new onset diabetes after transplantation; Bold: main variables and *p*-values.

**Table 3:** Mean eGFR and UPCR levels one year depending on the antihypertensive agent.

Antihypertensive Agent	Mean eGFR (mL/min/1.73 m^2^)	Mean UPCR (mg/g Creatinine)
ACE-I/ARB use (*n* = 443)	51.4 ± 19.5	289 ± 758
Beta-blocker use (*n* = 582)	50.5 ± 19.9	304 ± 818
Loop diuretic use (*n* = 331)	44.4 ± 17.6	417 ± 1049
CCB use (*n* = 440)	51.3 ± 20.5	306 ± 715

Abbreviations: DGF: delayed graft function; eGFR: estimated glomerular filtration rate; UPCR: urine protein/creatinine ratio.

**Table 4:** Multivariable Cox regression for factors associated with death-censored graft failure.

Variable	Hazard Ratio	95% CI	*p* Value
Age at KTx	0.980	0.953–1.008	**0.161**
Previous KTx	0.514	0.151–1.744	**0.285**
CMV-DNAemia	1.576	0.842–2.950	**0.155**
BKV-DNAemia	1.279	0.650–2.517	**0.476**
Post-mortal donation	0.411	0.116–1.464	**0.170**
Donor age	1.023	0.992–1.055	**0.152**
Dialysis vintage	1.001	0.991–1.012	**0.842**
Cold ischemia time	1.104	1.021–1.195	**0.014**
Delayed graft function	0.969	0.477–1.966	**0.930**
Acute rejection	4.428	2.249–8.720	**<0.001**
Beta-blocker use	0.884	0.305–2.561	**0.820**
Loop diuretic use	1.388	0.643–2.995	**0.404**
CCB use	0.604	0.248–1.468	**0.266**
ACE-I/ARB	0.619	0.278–1.379	**0.240**
Number of antihypertensives after 12 months	1.585	1.061–2.367	**0.025**
Underlying renal disease	-	-	**0.764**

Abbreviations: KTx: kidney transplantation; CMV: cytomegalovirus; BKV: BK-Polyomavirus; CCB: calcium channel blocker; ACE-I: Angiotensin-converting enzyme inhibitor; ARB: Angiotensin 1-receptor blocker. Bold: *p*-values.

**Table 5:** eGFR-change depended on the number of antihypertensive agents.

Variable	All	No Antihypertensive Drugs	1–2 Antihypertensive Drugs	3–4 Antihypertensive Drugs	≥5 Antihypertensive Drugs	*p* Value
eGFR-change year 1–2, mL/min/1.73 m² (Mean, SD)	0.31 ± 10.2	2.2 (12.6)	−0.17 (10.5)	0.9 (10.1)	1.8 (7.4)	0.405 *
eGFR-change year 1–5, mL/min/1.73 m² (Mean, SD)	2.4 (16.3)	−6.6 (15.6)	3.7 (15.7)	3.0 (16.9)	−1.8 (15.4)	0.032 *

* Kruskal-Wallis-test, Abbreviations: eGFR: estimated glomerular filtration rate; SD: standard deviation. Bold: *p* values

## Supplementary Materials

The following are available online at https://www.mdpi.com/2077-0383/9/12/3969/s1, Figure S4A–D: Unadjusted survival plots for different drug classes.
