# Supplementary material for: Antihypertensive Treatment in Kidney Transplant Recipients—A Current Single Center Experience
Source: J Clin Med. 2020 Dec 7;9(12):3969. doi: 10.3390/jcm9123969 (PMC7762385; doi:10.3390/jcm9123969)
Supplement: Supplementary file 1 [file jcm-09-03969-s001.pdf]

Journal of
Clinical Medicine

MDPI

Supplementary Materials: The following are available online at www.mdpi.com/2077-0383/9/12/3969/s1

Unadjusted survival plots for different drug classes

Supplementary Figure S4A:

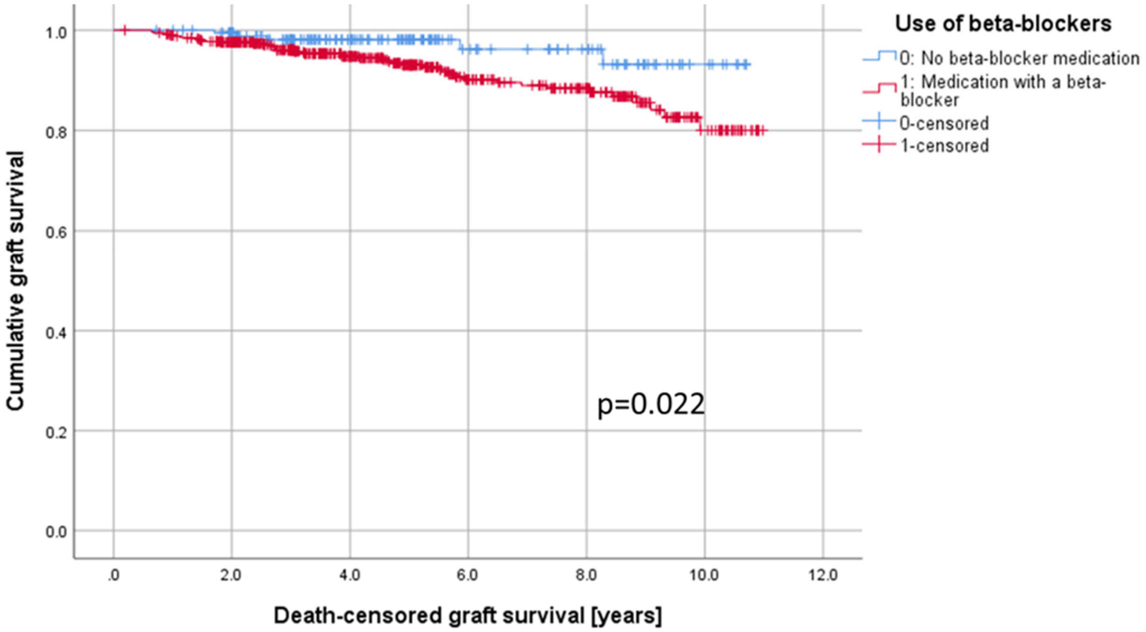

Patients-at-risk

No beta-blockers	182	165	98	49	37	9	0
Med. with beta-blockers	581	522	320	170	122	30	0

Supplementary Figure S4B:

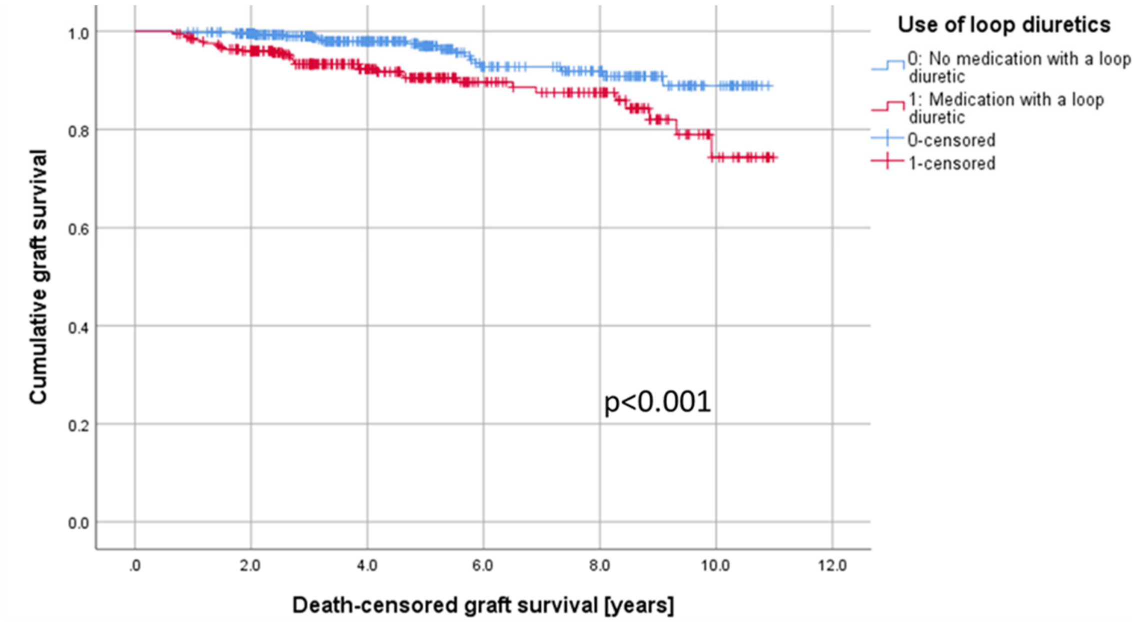

Patients-at-risk

No loop diuretics	423	384	238	124	90	24	0
Med. with loop diuretic	331	295	174	91	66	15	0

Supplementary Figure S4C:

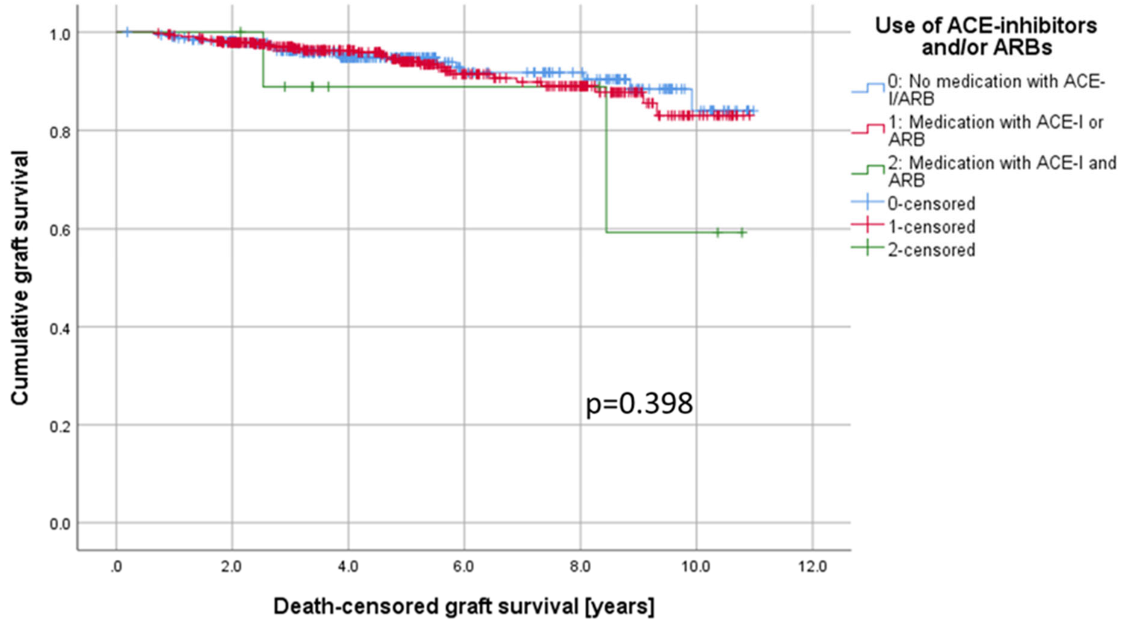

Patients-at-risk

No ACE-I/ARB	322	289	180	86	67	19	0
Med. with ACE-I/ARB	432	388	234	129	88	18	0
Med. with ACE-I/ARB	10	10	4	4	4	1	0

Supplementary Figure S4D:

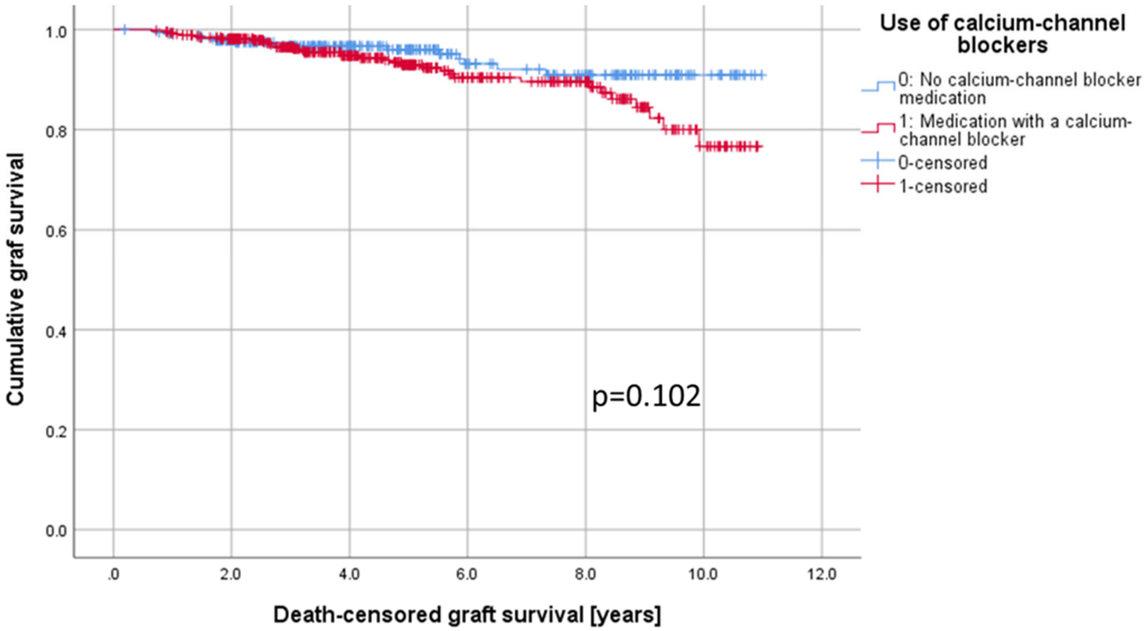

Patients-at-risk

No CCB	325	286	174	92	69	17	0
Med. with CCB	439	401	244	127	90	22	0

Suppl. Figure 4: Patients treated with beta-blockers show unfavorable death-censored graft survival compared to those without, Log-rank test, p=0.022 **(A)**. Those treated with loop diuretics also show unfavorable death-censored graft survival compared to those without, Log-rank test, p=0.001 **(B)**. In contrast, the use of ACE-I/ARBs does not influence death censored allograft survival, Log-rank test, p=0.398 **(C)**. Likewise, there is no difference in the death-censored allograft survival between patients with and without CCB medication, Log-rank test, p=0.102 **(D)**
